# Supplementary material for: Horizontal acquisition of a DNA ligase improves DNA damage tolerance in eukaryotes
Source: Nat Commun. 2023 Nov 22;14:7638. doi: 10.1038/s41467-023-43075-8 (PMC10665377; doi:10.1038/s41467-023-43075-8)
Supplement: Supplementary file 1 — Supplementary Information [file 41467_2023_43075_MOESM1_ESM.pdf]

Supplementary Information for

Horizontal acquisition of a DNA ligase improves DNA damage tolerance in eukaryotes

Emilien Nicolas[1,*], Paul Simion[2,3], Marc Guérineau[1], Matthieu Terwagne[2], Mathilde Colinet[2], Julie Virgo[2], Maxime Lingurski[1], Anaïs Boutsen[2], Marc Dieu[4], Bernard Hallet[5,*], and Karine Van Doninck[1,2,*]

[1]Université Libre de Bruxelles, Molecular Biology and Evolution, Brussels, 1050, Belgium
[2]Université de Namur, Laboratory of Evolutionary Genetics and Ecology, Namur, 5000, Belgium
[3]Université de Rennes 1, Ecosystèmes, biodiversité, évolution (ECOBIO), CNRS, Rennes, France
[4]Université de Namur, MaSUN-mass spectrometry facility, Namur, 5000, Belgium
[5]Université Catholique de Louvain, Louvain Institute of Biomolecular Science and Technology, Louvain-la-Neuve, 1348, Belgium
[*]Corresponding authors: emilien.nicolas@gmail.com, bernard.hallet@uclouvain.be, karine.van.doninck@ulb.be

Table of Contents

Supplementary Figures 3

Figure S1. Upregulation of a DNA ligase upon irradiation of the bdelloid rotifer *Adineta vaga* 3

Figure S2. Horizontal acquisition of DNA ligase E by *Adineta vaga* 6

Supplementary Tables 7

Table S1. Mass spectrometry analysis. Representation of the proteins that are over/under-represented more or equal than 2.5 times in comparison to non-irradiated controls after irradiation with X-Rays 7

Table S2. Alienomics scores 10

Table S3. List of species included in the phylogenetic analysis 10

Table S4. List of oligonucleotides used in this study 15

Source data files for supplementary information 19

Supplementary Figures

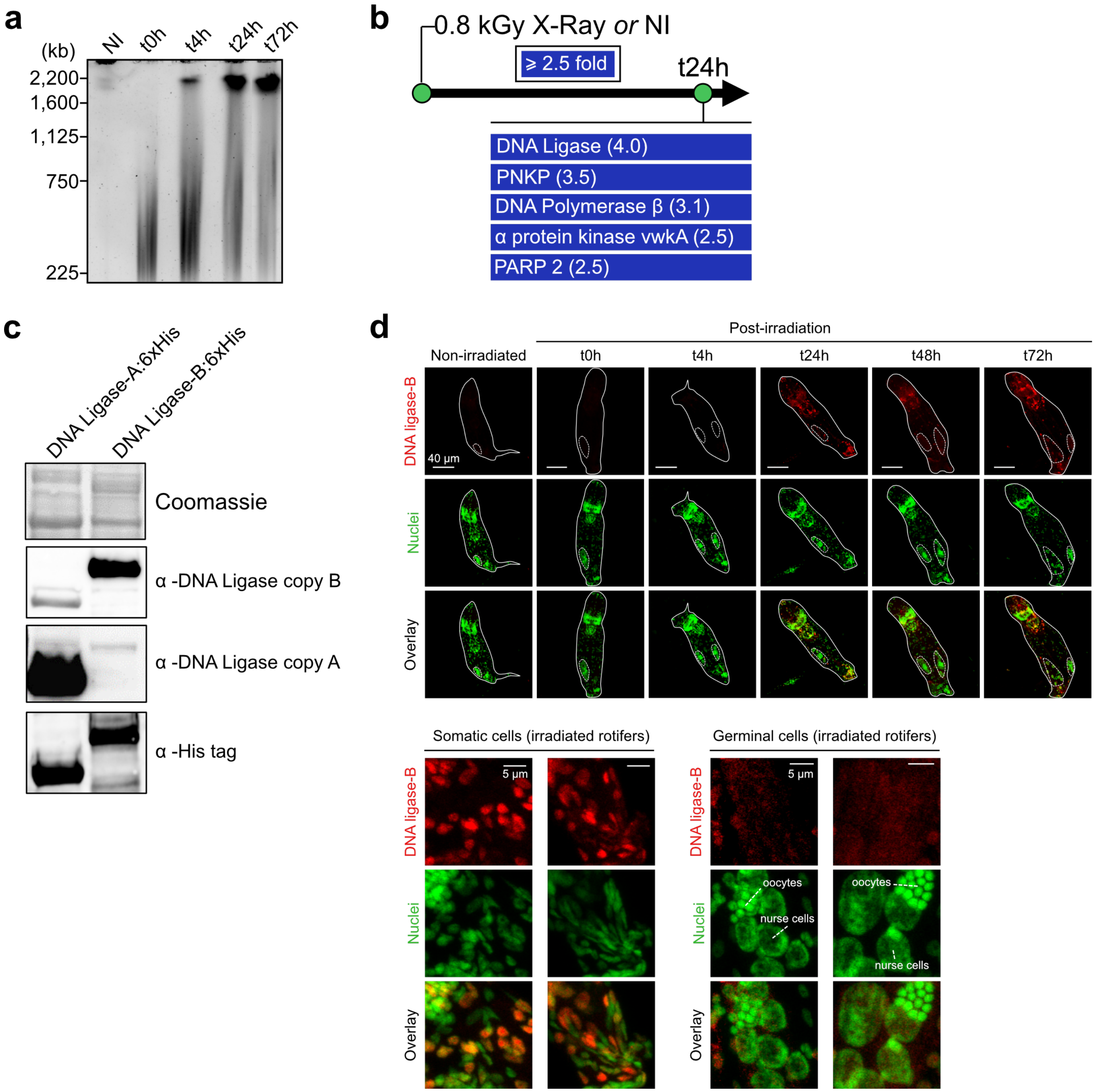

Figure S1. Upregulation of a DNA ligase upon irradiation of the bdelloid rotifer *Adineta vaga*. a. PFGE image showing the kinetics of DNA DSB repair upon irradiation at an X-ray dose of 1 kGy. The smear remaining at late time points (t24h or t72h) suggests an incomplete DNA repair, leaving unassembled genomic fragments. The weak signal observed for the NI samples likely results from the fact that intact *A. vaga* chromosomes are too big to enter into the gel. **b**. Global proteomic analysis at 0.8 kGy of X-ray. Total protein were extracted from the rotifers at t24h post-irradiation. Differential expression analysis of each protein was performed with Peaks studio. This scheme shows the proteins that are over-represented (≥ 2.5x) in comparison to the non-irradiated control (NI). **c**. Western-blot analysis showing the efficiency of the polyclonal antibodies

targeting the copies A or B of the DNA ligase E of *A. vaga*. The recombinant proteins were expressed in *E. coli*. After induction, the total protein extracts were separated on a SDS-PAGE gel and submitted to Western-blot analysis with antibodies targeting DNA Ligase E copy B, DNA Ligase E copy A, or the poly-histidine tag. **d.** Subcellular localization analysis of the induced DNA ligase E (copy B) by immuno-fluorescence at different timepoints post-irradiation (replicate of Figure 1C). White lines delineate the bdelloid rotifer *A. vaga* individual and dotted lines delimit the ovaries of *A. vaga* in the middle part of their body, where less to no staining of DNA ligase-B was observed. Enlargements on somatic and germinal cells show the difference in DNA Ligase-B localization between both cell types. Nuclei were stained with DAPI but were displayed in green to ease the interpretation of the result.

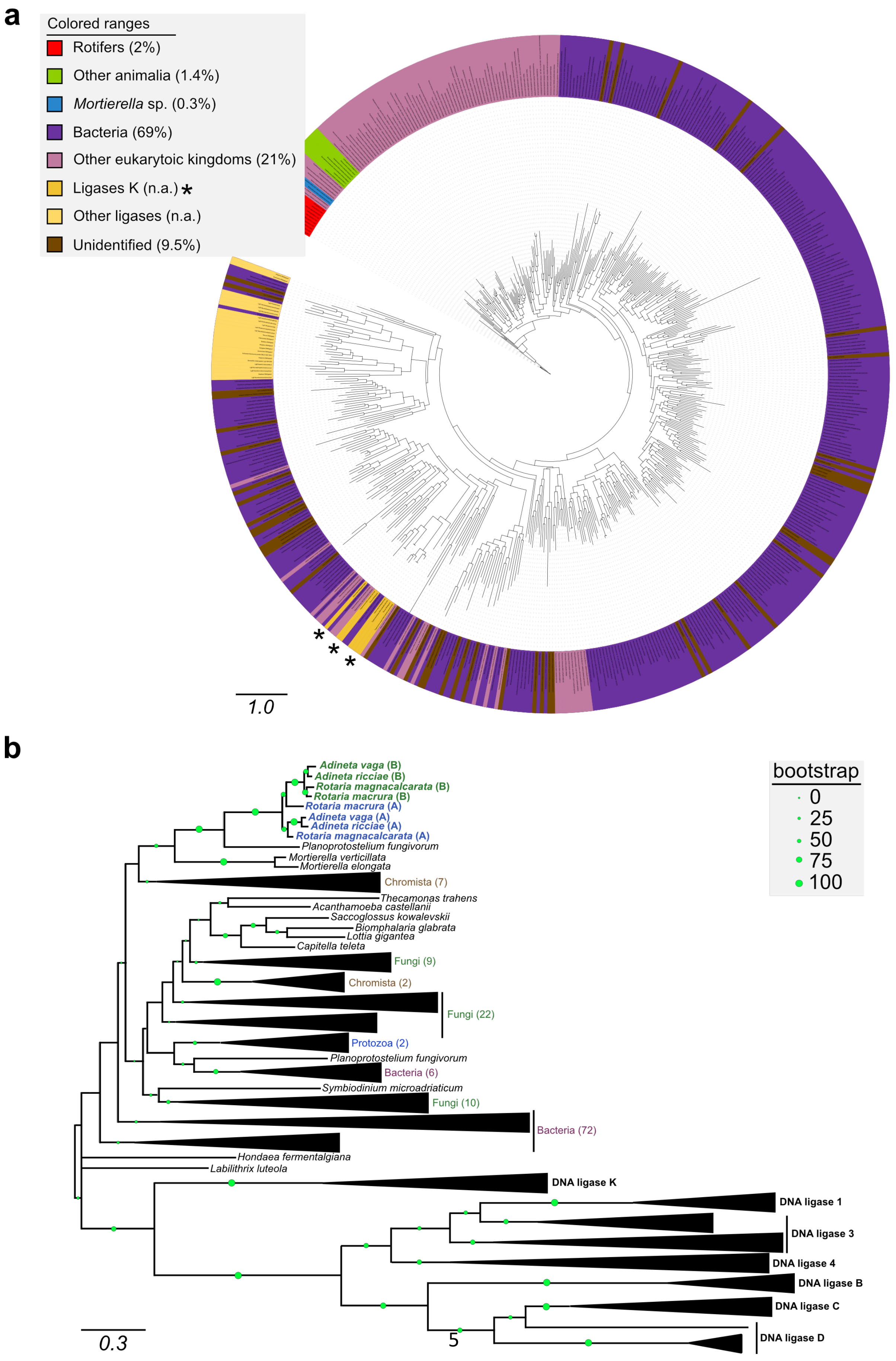
a
Colored ranges
Rotifers (2%)
Other animalia (1.4%)
Mortierella sp. (0.3%)
Bacteria (69%)
Other eukarytoic kingdoms (21%)
Ligases K (n.a.) *
Other ligases (n.a.)
Unidentified (9.5%)
* * *
1.0
b
bootstrap
0
25
50
75
100
Adineta vaga (B)
Adineta ricciae (B)
Rotaria magnacalcarata (B)
Rotaria macrura (B)
Rotaria macrura (A)
Adineta vaga (A)
Adineta ricciae (A)
Rotaria magnacalcarata (A)
Planoprotostelium fungivorum
Mortierella verticillata
Mortierella elongata
Chromista (7)
Thecamonas trahens
Acanthamoeba castellanii
Saccoglossus kowalevskii
Biomphalaria glabrata
Lottia gigantea
Capitella teleta
Fungi (9)
Chromista (2)
Fungi (22)
Protozoa (2)
Planoprotostelium fungivorum
Bacteria (6)
Symbiodinium microadriaticum
Fungi (10)
Bacteria (72)
Hondaea fermentalgiana
Labilithrix luteola
DNA ligase K
DNA ligase 1
DNA ligase 3
DNA ligase 4
DNA ligase B
DNA ligase C
DNA ligase D
0.3

Figure S2. Horizontal acquisition of DNA ligase E by *Adineta vaga*. a. Phylogenetic analysis of the DNA ligase over-represented in *A. vaga* following exposure to X-ray. This circular tree is the full taxonomic sampling including 502 hits retrieved from the protein blast analysis against uniref50 database. The relative representations of taxonomic groups are represented. n.a. = not applicable because the selected sequences are just a subset from these families of proteins. **b.** Phylogenetic analysis performed with the ATP-dependent DNA ligase and OB-fold domains alone. The numbers between brackets represent the number of species from the same kingdom that have been collapsed.

Supplementary Tables

**Table S1. Mass spectrometry analysis. Representation of the proteins that are over/under-represented more or equal than 2.5 times in comparison to non-irradiated controls after irradiation with X-Rays**								
**1.0 kGy of irradiation**								
**Time after irradia-tion**	**Protein Accession**	**BLASTp result**	**Potential function**	**Over-/Under-representa-tion**	**Group Profile (Ratio)**	**Cove-rage (%)**	**#Pep-tides**	**#Uni-que**
t0h	/	/	/	/	/	/	/	/
t4h	UJR14855.1	DNA Ligase	DNA replication, DNA repair	Over-	1.00: 6.29	22	7	7
	UJR34273.1	DNA Polymerase beta	DNA replication, DNA repair	Over-	1.00: 3.95	15	3	3
	UJR16087.1	DNA 3' Phosphatase (PNKP)	DNA repair	Over-	1.00: 3.88	52	17	17
	UJR36762.1	PARP domain-containing protein	DNA repair, genomic stability, and programmed cell death	Over-	1.00: 2.92	7	2	2
	UJR37663.1	Alpha-protein kinase vwka	Muscle contraction	Over-	1.00: 2.75	7	3	3
	UJR15000.1	Poly [ADP-ribose] polymerase 2 (PARP2)	DNA repair, genomic stability, and programmed cell death	Over-	1.00: 2.47	25	12	11
t24h	UJR14855.1	DNA Ligase	DNA replication, DNA repair	Over-	1.00: 6.72	20	6	6
	UJR16087.1	DNA 3' Phosphatase (PNKP)	DNA repair	Over-	1.00: 4.37	52	17	17
	UJR34273.1	DNA Polymerase beta	DNA replication, DNA repair	Over-	1.00: 3.92	15	3	3
	UJR37663.1	Alpha-protein kinase vwka	Muscle contraction	Over-	1.00: 3.29	10	4	4
	UJR21294.1	AAA family ATPase	Diverse cellular activities	Over-	1.00: 3.11	35	10	9
	UJR15000.1	Poly [ADP-ribose]	DNA repair, genomic	Over-	1.00: 2.91	23	11	10

		polymerase 2 (PARP2)	stability, and programmed cell death					
	UJR35110.1	Unknown protein product	Unknown function	Over-	1.00: 2.46	19	3	3
	UJR22637.1	40s ribosomal protein s4	Ribosome component	Under-	1.00: 0.32	39	11	2
t72h	UJR14855.1	DNA Ligase	DNA replication, DNA repair	Over-	1.00: 6.07	24	8	7
	UJR34273.1	DNA Polymerase beta	DNA replication, DNA repair	Over-	1.00: 5.16	22	4	4
	UJR16087.1	DNA 3' Phosphatase (PNKP)	DNA repair	Over-	1.00: 4.72	37	13	13
	UJR21294.1	AAA family ATPase	Diverse cellular activities	Over-	1.00: 3.87	36	11	10
	UJR37663.1	Alpha-protein kinase vwka	Muscle contraction	Over-	1.00: 3.50	10	4	4
	UJR27866.1	Post-GPI attachment to proteins factor 3 (PGAP3)	Lipid remodeling step of GPI-anchor maturation.	Over-	1.00: 3.36	15	8	8
	UJR15000.1	Poly [ADP-ribose] polymerase 2 (PARP2)	DNA repair, genomic stability, and programmed cell death	Over-	1.00: 3.35	26	13	12
	UJR23261.1	Conserved Plasmodium protein	Unknown function	Over-	1.00: 3.06	15	6	2
	UJR35110.1	Unknown protein product	Unknown function	Over-	1.00: 2.85	25	4	4
	UJR11436.1	NAD-dependent protein deacetylase srt1	Epigenetic regulation during DNA damage	Over-	1.00: 2.55	58	11	11
	UJR33709.1	Carrier protein mitochondrial-like	Mitochondrial metabolite transport	Under-	1.00: 0.39	30	9	3
	UJR21766.1	Methyltransferase	Diverse cellular activities	Under-	1.00: 0.39	20	5	3
	UJR13421.1	Translocon-associated protein	Recycling of the translocation	Under-	1.00: 0.38	9	2	2

			apparatus at the ER					
	UJR12117.1	group XIIA secretory phospholipase A2	Phospholipid metabolism	Under-	1.00: 0.38	19	2	2
	UJR09444.1	voltage-dependent anion-selective channel protein 2	Cell metabolism	Under-	1.00: 0.37	15	3	2
	UJR38387.1	Glycoside hydrolase	Hydrolysis of glycosidic bonds in complex sugars	Under-	1.00: 0.37	13	2	2
	UJR38099.1	Cyclophilin b	Cell metabolism, energy homeostasis	Under-	1.00: 0.35	19	3	3
	UJR15177.1	Microsomal glutathione S-transferase 1	Oxidative stress protection	Under-	1.00: 0.33	21	3	3
	UJR22749.1	Protein dcd1a-like	Lipid metabolism	Under-	1.00: 0.30	28	8	7
	UJR22637.1	40s ribosomal protein x isoform	Ribosome component	Under-	1.00: 0.26	39	10	2
**0.8 kGy of irradiation**								
**Time after irradia-tion**	**Protein Accession**	**BLASTp result**	**Potential function**	**Over-/Under-representa-tion**	**Group Profile (Ratio)**	**Cove-rage (%)**	**#Pep-tides**	**#Uni-que**
t24h	UJR14855.1	DNA Ligase	DNA replication, DNA repair	Over-	1.00: 4.04	34	12	11
	UJR16087.1	DNA 3' Phosphatase (PNKP)	DNA repair	Over-	1.00: 3.45	43	12	12
	UJR34273.1	DNA Polymerase beta	DNA replication, DNA repair	Over-	1.00: 3.06	11	3	3
	UJR37663.1	Alpha-protein kinase vwka	Muscle contraction	Over-	1.00: 2.53	5	2	2
	UJR15000.1	Poly [ADP-ribose] polymerase 2 (PARP2)	DNA repair, genomic stability, and programmed cell death	Over-	1.00: 2.46	34	17	16

This table represents the accession number and description of potential functions of the proteins over- and under-represented after X-Ray exposure of the bdelloid rotifer *Adineta vaga*. The Group Profile (Ratio) represents the fold change in the quantity of at least two peptides per protein in comparision to their relative amount in the non-irradiated condition. Coverage (%) is the coverage of all detected peptides on the full sequence of the different proteins. #Peptides is the number of peptides detected for each protein and #Unique represents the number of unique, non-overlapping, peptides detected for each protein.

Table S2. Alienomics scores

gene_ID	Scaf-fold	Catego-ry	start	end	BLAST score	GC score (%GC)	coverage score	expression score (TPM)
UJR14855.1	Chrom_1	expressed HGT	5793041	5794788	-0.977	0 (35.1%)	0	1 (2.4)
UJR31854.1	Chrom_4	expressed HGT	7344188	7345745	-0.999	0 (34.7%)	0	1 (3.5)

BLAST scores around -1 (the smallest possible score) represent very strong best-hit on non-metazoan sequences; coverage score at 0 indicates that read coverage is the same as the average read coverage observed along the genome (indicating that the gene is integrated within *A. vaga* chromosomes); expression score of 1 indicates that this gene is expressed in hydrated condition (based on RNA-Seq data) though at a very low level (2.4 and 3.5 transcripts per million, respectively). Finally, a GC score of 0 indicates that the GC content of these Ligase E genes is not sufficiently different (35%) from the GC content observed in the rest of the genome (31%) to solely suggest an HGT origin.

Table S3. List of species included in the phylogenetic analysis

Species name	Kingdom*	Sequence code	
Adineta vaga	Animalia	UJR14855.1	
Adineta ricciae	Animalia	g39941.t1:7261()	
Rotaria magnacalcarata	Animalia	g35086.t1:7261()	
Rotaria macrura	Animalia	g8068.t1:7261()	
Rotaria macrura	Animalia	g6157.t1:4262()	
Adineta vaga	Animalia	UJR31854.1	
Adineta ricciae	Animalia	g54777.t1:4262()	
Rotaria magnacalcarata	Animalia	g31225.t1:7262()	
Planoprotostelium fungivorum	Protozoa	UniRef50_A0A2P6NS38	
Mortierella verticillata	Fungi	UniRef50_A0A086TKT4	
Mortierella elongata	Fungi	UniRef50_A0A197JFC3	
Stentor coeruleus	Chromista	UniRef50_A0A1R2CH16	
Paramecium tetraurelia	Chromista	UniRef50_A0CCG0	Group 1
Tetrahymena thermophila	Chromista	UniRef50_Q233G4	
Ichthyophthirius multifiliis	Chromista	UniRef50_G0QN32	
Pseudocohnilembus persalinus	Chromista	UniRef50_A0A0V0R6G1	
Oxytricha trifallax	Chromista	UniRef50_J9IMQ8	
Stylonychia lemnae	Chromista	UniRef50_A0A078AES1	
Symbiodinium microadriaticum	Chromista	UniRef50_A0A1Q9DVN9	
Planoprotostelium fungivorum	Protozoa	UniRef50_A0A2P6NC06	

Acanthamoeba castellanii	Protozoa	UniRef50_L8H8E1	
Saccoglossus kowalevskii	Animalia	UniRef50_UPI0001CBB244	
Lottia gigantea	Animalia	UniRef50_V3ZA01	
Biomphalaria glabrata	Animalia	UniRef50_A0A2C9L857	
Capitella teleta	Animalia	UniRef50_R7T7U7	
Hondaea fermentalgiana	Chromista	UniRef50_A0A2R5GGV1	
Allomyces macrogynus	Fungi	UniRef50_A0A0L0S6H7	Group 2
Catenaria anguillulae	Fungi	UniRef50_A0A1Y2HSF4	
Rhizophagus irregularis	Fungi	UniRef50_U9U428	
Diversispora epigaea	Fungi	UniRef50_A0A397HNX5	
Endogone sp.	Fungi	UniRef50_A0A433PPJ8	
Phytophthora parasitica	Fungi	UniRef50_W2QYG0	
Phytophthora kernoviae	Fungi	UniRef50_A0A3R7G5I6	
Pythium insidiosum	Fungi	UniRef50_A0A2D4BL39	
Bifiguratus adelaidae	Fungi	UniRef50_A0A261XUB6	
Serendipita vermifera	Fungi	UniRef50_A0A0C2WQ68	Group 3
Salpingoeca rosetta	Fungi	UniRef50_F2UAM4	
Powellomyces hirtus	Fungi	UniRef50_A0A507EFK5	
Spizellomyces sp.	Fungi	UniRef50_A0A507F3V8	
Chytriomyces confervae	Fungi	UniRef50_A0A507FML9	
Punctularia strigosozonata	Fungi	UniRef50_R7S2Z7	
Neolentinus lepideus	Fungi	UniRef50_A0A165WCR3	
Schizophyllum commune	Fungi	UniRef50_D8PMU8	
Laccaria bicolor	Fungi	UniRef50_B0DDS4	
Galerina marginata	Fungi	UniRef50_A0A067TX69	
Coprinopsis cinerea	Fungi	UniRef50_A8P6S5	
Moniliophthora roreri	Fungi	UniRef50_V2YHU7	
Coprinellus micaceus	Fungi	UniRef50_A0A4Y7TF26	
Thanatephorus cucumeris	Fungi	UniRef50_A0A0B7FYV4	
Dactylellina haptotyla	Fungi	UniRef50_S8C735	
Pyronema omphalodes	Fungi	UniRef50_U4LWK6	
Tuber borchii	Fungi	UniRef50_A0A2T6ZFH0	
Tuber melanosporum	Fungi	UniRef50_D5GLS4	
Ascobolus immersus	Fungi	UniRef50_A0A3N4I9N9	
Terfezia boudieri	Fungi	UniRef50_A0A3N4M3N8	
Aphanomyces stellatus	Chromista	UniRef50_A0A485LIR3	Group 4
Saprolegnia diclina	Chromista	UniRef50_T0PU85	
Thecamonas trahens	Protozoa	UniRef50_A0A0L0DPB3	
Tilletia walkeri	Fungi	UniRef50_A0A177V150	Group 5
Tilletia controversa	Fungi	UniRef50_A0A177VL16	
Acaromyces ingoldii	Fungi	UniRef50_A0A316YL01	

Sporisorium graminicola	Fungi	UniRef50_A0A4U7KRU1	
Malassezia globosa	Fungi	UniRef50_A8Q7S3	
Tilletiaria anomala	Fungi	UniRef50_A0A066VK17	
Rhodotorula taiwanensis	Fungi	UniRef50_A0A2S5BI12	
Rhodotorula graminis	Fungi	UniRef50_A0A0N8Q036	
Leucosporidium creatinivorum	Fungi	UniRef50_A0A1Y2FRX1	
Microbotryum lychnidis-dioicae	Fungi	UniRef50_U5HDG7	
Capsaspora owczarzaki	Protozoa	UniRef50_A0A0D2WMT9	Group 6
Bodo saltans	Protozoa	UniRef50_A0A0S4IPC3	
Blastopirellula marina	Bacteria	UniRef50_A4A2I8	Group 7
Singulisphaera sp.	Bacteria	UniRef50_A0A1N6KPA9	
Myxococcus xanthus	Bacteria	UniRef50_Q1DEP0	
Chondromyces crocatus	Bacteria	UniRef50_A0A0K1EBB1	
Nannocystis exedens	Bacteria	UniRef50_A0A1I2CEZ6	
Enhygromyxa salina	Bacteria	UniRef50_A0A0C2D1E1	
Verrucomicrobiales bacterium	Bacteria	UniRef50_A0A2E5ELT1	Group 8
Verrucomicrobiales bacterium	Bacteria	UniRef50_A0A2V5L8T4	
Pelagibaculum spongiae	Bacteria	UniRef50_A0A2V1H2B4	
Hydrothermal vent metagenome	Bacteria	UniRef50_A0A3B1A5H6	
Aliikangiella marina	Bacteria	UniRef50_A0A545TEB3	
Gammaproteobacteria bacterium	Bacteria	UniRef50_A0A2A4W1R8	
Thiohalocapsa sp.	Bacteria	UniRef50_V4JW06	
Marinobacter sp.	Bacteria	UniRef50_U7GE35	
Marine sediment metagenome	Bacteria	UniRef50_A0A0F9N5V7	
Marinobacter sp.	Bacteria	UniRef50_A0A0P7WT61	
Catenovulum sp.	Bacteria	UniRef50_A0A0J8GSS8	
Neiella marina	Bacteria	UniRef50_UPI000B3D0BD5	
Chromatiaceae	Bacteria	UniRef50_A0A0M0M502	
Shewanella sp.	Bacteria	UniRef50_A0A0Q0GG30	
Parashewanella curva	Bacteria	UniRef50_A0A3L8PVI3	
Shewanella psychrophila	Bacteria	UniRef50_A0A1S6HLY7	
Thalassotalea crassostreae	Bacteria	UniRef50_UPI000839620A	
Colwellia sp.	Bacteria	UniRef50_A0A1D8RX27	
Thalassotalea sp.	Bacteria	UniRef50_A0A1Q6CHX0	
Thalassomonas sp.	Bacteria	UniRef50_A0A553F9N6	
Psychromonas ingrahamii	Bacteria	UniRef50_A1SU29	
Alteromonadales bacterium	Bacteria	UniRef50_A0A2N0WLJ3	
Methylomicrobium alcaliphilum	Bacteria	UniRef50_G4SZS2	
Gammaproteobacteria bacterium	Bacteria	UniRef50_F3KBF9	
Thiotrichales bacterium	Bacteria	UniRef50_A0A1H6FJG3	
Catenovulum sediminis	Bacteria	UniRef50_UPI001180CAE8	

Arsukibacterium sp.	Bacteria	UniRef50_A0A0M2VMP9	
Deltaproteobacteria bacterium	Bacteria	UniRef50_A0A355HBC5	
Amphritea sp.	Bacteria	UniRef50_A0A430KN85	
Amphritea balenae	Bacteria	UniRef50_A0A3P1SXF4	
Leucothrix arctica	Bacteria	UniRef50_A0A317C9N5	
Cocleimonas flava	Bacteria	UniRef50_A0A4R1ETL2	
Cellvibrionaceae bacterium	Bacteria	UniRef50_A0A2E2XPW1	
Collimonas fungivorans	Bacteria	UniRef50_G0ACU4	
Burkholderiales bacterium	Bacteria	UniRef50_A0A257FHV3	
Uncultured bacterium	Bacteria	UniRef50_A0A0K2VM40	
Stenotrophomonas acidaminiphila	Bacteria	UniRef50_A0A0S1AZ25	
Alphaproteobacteria bacterium	Bacteria	UniRef50_A0A1V6FG67	
Acidovorax sp.	Bacteria	UniRef50_A1W9H7	
Variovorax sp.	Bacteria	UniRef50_A0A1H7HCH0	
Curvibacter sp.	Bacteria	UniRef50_A0A1Y0N947	
Marinagarivorans algicola	Bacteria	UniRef50_UPI0009E90CBC	
Oleiphilus messinensis	Bacteria	UniRef50_A0A1H5SE61	
Vibrio sp.	Bacteria	UniRef50_A0A3D1Z3Q8	
Vibrio algivorus	Bacteria	UniRef50_A0A557P7I3	
Vibrio caribbeanicus	Bacteria	UniRef50_E3BFM3	
Aliivibrio	Bacteria	UniRef50_A0A1B9PDH1	
Vibrio tapetis	Bacteria	UniRef50_A0A2N8Z9V5	
Salinivibrio kushneri	Bacteria	UniRef50_A0A4P8H0E3	
Marinobacterium jannaschii	Bacteria	UniRef50_UPI0004842EBE	
Oceanisphaera avium	Bacteria	UniRef50_A0A1Y0CUY0	
Ferrimonas sediminum	Bacteria	UniRef50_A0A1G8TBD7	
Aliagarivorans sp.	Bacteria	UniRef50_UPI0003F8BE47	
Agarivorans albus	Bacteria	UniRef50_R9PLS9	
Gallaecimonas xiamenensis	Bacteria	UniRef50_K2J486	
Candidatus Entotheonella factor	Bacteria	UniRef50_W4LW30	
Candidatus Entotheonella gemina	Bacteria	UniRef50_W4LJW3	
Candidatus Magnetomorum	Bacteria	UniRef50_A0A0M9ECM2	
Zetaproteobacteria bacterium	Bacteria	UniRef50_A0A2M7PYS4	
Deltaproteobacteria bacterium	Bacteria	UniRef50_A0A354FJP9	
Desulfuromonadales	Bacteria	UniRef50_A0A1F9Q5P5	
Desulfuromonas sp.	Bacteria	UniRef50_A0A3B8ZZN3	
Desulfuromonas sp.	Bacteria	UniRef50_A0A143B629	
Sedimenticola thiotaurini	Bacteria	UniRef50_A0A0F7K268	
Hydrogenovibrio crunogenus	Bacteria	UniRef50_A0A4P7P0Z1	
Campylobacter ureolyticus	Bacteria	UniRef50_A0A0K1HH14	
Campylobacter curvus	Bacteria	UniRef50_A7GWV3	

Sulfurospirillum	Bacteria	UniRef50_W6EGB2	
Sulfurimonas gotlandica	Bacteria	UniRef50_B6BKK3	
Candidatus Kentron	Bacteria	UniRef50_A0A451B8H6	
Spizellomyces punctatus	Fungi	UniRef50_A0A0L0HLR9	Group 9
Powellomyces hirtus	Fungi	UniRef50_A0A507E4I8	
Labilithrix luteola	Bacteria	UniRef50_A0A0K1PJR5	
Gammaproteobacteria bacterium	Bacteria	UniRef50_A0A293NAA4	
Chromera velia	Chromista	UniRef50_A0A0G4GGN3	
*Trypanosoma brucei***	Protozoa	Q6V9I8_9TRYP	Group 10 (LIGK)
*Crithidia fasciculata***	Protozoa	Q53E04_CRIFA	
*Trypanosoma brucei***	Protozoa	D6XJX8_TRYB2	
*Crithidia fasciculata***	Protozoa	Q6U1Z6_CRIFA	
*Adineta vaga***	Animalia	UJR07604.1	Group 11 (Ligase 1)
*Adineta vaga***	Animalia	UJR07606.1	
*Homo sapiens***	Animalia	P18858 (DNLI1_HUMAN)	
*Caenorhabditis elegans***	Animalia	Q27474 (DNLI1_CAEEL)	
*Saccharomyces cerevisiae***	Fungi	P04819 (DNLI1_YEAST)	
*Trypanosoma brucei***	Protozoa	Q587E4_TRYB2	
*Crithidia fasciculata***	Protozoa	Q23694_CRIFA	
*Bodo saltans***	Protozoa	A0A0S4JHN9_BODSA	
*Schizosaccharomyces pombe***	Fungi	Q9C1W9 (DNLI3_SCHPO)	Group 12 (Ligase 3)
*Adineta vaga***	Animalia	UJR26750.1	
*Homo sapiens***	Animalia	P49916 (DNLI3_HUMAN)	
*Drosophila melanogaster***	Animalia	Q9VG84_DROME	
*Adineta vaga***	Animalia	UJR20821.1	Group 13 (Ligase 4)
*Homo sapiens***	Animalia	P49917 (DNLI4_HUMAN)	
*Saccharomyces cerevisiae***	Fungi	Q08387 (DNLI4_YEAST)	
*Bodo saltans***	Protozoa	A0A0S4IX10_BODSA	
*Mycobacterium tuberculosis***	Bacteria	P9WNV5 (DNLI_MYCTU)	Group 14 (Ligase B)
*Aquamicrobium defluvii***	Bacteria	A0A011TCU6_9RHIZ	
*Novosphingobium resinovorum***	Bacteria	A0A031JZ36_9SPHN	
*Rubellimicrobium mesophilum***	Bacteria	A0A017HNQ3_9RHOB	
*Mycobacterium tuberculosis***	Bacteria	A0A109SB90_MYCBI	Group 15 (Ligase C)
Streptomyces sp.**	Bacteria	A0A022MQ55_9ACTN	
*Rhodococcus aetherivorans***	Bacteria	A0A059MI09_9NOCA	
*Saccharopolyspora rectivirgula***	Bacteria	A0A073B9T4_9PSEU	
*Mycolicibacterium smegmatis***	Bacteria	A0A653F9L4_MYCSM	Group 16 (Ligase D)
*Mycobacterium tuberculosis***	Bacteria	P9WNV3 (LIGD_MYCTU)	
Pedobacter sp.**	Bacteria	A0A520IIW8_9SPHI	
*Pseudomonas aeruginosa***	Bacteria	Q9I1X7 (LIGD_PSEAE)	
*Burkholderia multivorans***	Bacteria	WP_164675907.1	

*, based on classification from Ruggiero, M. A. *et al*. A higher level classification of all living organisms. PLoS One 10, e0119248 (2015).
**, sequences that have been added independently of the blastp analysis for the phylogenetic analysis

Table S4. List of oligonucleotides used in this study

Orga-nism	Target gene	Accession number	Name	Sequence (5'>3')	Source
Adineta vaga	*gapdh*	FUN_010333	Avaga_GAPDH_FW	TGTGCTGCAATCAAAGAAGC	1
			Avaga_GAPDH_RV	CGACACGGTTTGAATAACCA	
	DNA Ligase E_homoeo-log B	FUN_001822	Avaga_LIGEb_FW1	CCACGATTTCCGACATTTCT	
			Avaga_LIGEb_RV1	CATCTTCGTCATCGGATGTG	
	DNA Ligase E_homoeo-log A	FUN_019185	Avaga_LIGEa_FW2	GCTGGTATTCCACGTTTTCC	
			Avaga_LIGEa_RV2	TCTTCATCGGATGCAACAAC	
	DNA Ligase E_homoeo-log B	FUN_001822	Avaga_gblock_LIGEb	AGCTCATGACGCGATGTGTAGTGAAGCTGCTAAAGTTCTTCTTGCTGAAACATGGTCAGAAGATATTGATCCGACAGGATGGTGGATGAGTGAAAAACTCGATGGTGTTCGAGCATACTGGAATGGAAAAAATTTTTATTCAAGACAAGGAAATCTTTTTCATGCACCTGATTTTTTCAAGGCTGCACTACCTAAAGTTCCACTCGATGGTGAAATATGGTGTGGCCGGGGTTTATTTCAAAAATGTGTTAGTATTGTCAAAAAACAAGCAAATAAAGTGGTGCCTCAAGATTATAAACTACTGACGTATCTAATTTTTGACGCACCAACTCAAGGAGGAAAATATGAAGATCGTGTCAAATGGTTACAAGCAAATATTCCACAAGATGATGATAACTGTTATGCTACAGTTGTTGGTATTAAAAAATGCGAAGGTTTAGCACAATTAAAACAATATTTAGCTGATGTGAACAAAGCTGGTGGAGAAGGTATTATGCTTCGTAAACCAGGTAGTCTTTATGAACATAAACGTTCAACTACTTTACTGAAAGTAAAAACGTTTTATGACGAAGAAGCTCATGTCATTGGTCATAAACCGAGTAAAAGCTTAATTGGTATGACTGGTGCTCTTGAATGTGAATTACCGAATGGAAAACGATTTGATGTTGGTAGTGGACTTACTATGGATCAACGACGAAAACCACCAAAAATCGGTTCAGTCATTACATTTAAATTTCAAGAATTATCCAATAGTGGTAGCCCACGATTTCCGACATTTCTTCGTGTTCGTACTGATCTGACATGGAATGATGTTCTTGAAGCAGCTAAAACAAAAAAACCTGTCAGTGTCATACAGAAAGTGGTTCCATCGGCAAAATTATCTAAACAACATTCAATATTATTTTCTGTTATTCCATCACGTGATGCCAAAACCGATAAGAAAATCGTCACATCCGATGACGAAGATGAAGATGCACCATCTACATCTACATCCACATCGACAAAGACGAATGATTCTCGACCGATGTGTAAATTTGGTGCAAAATGTTATCGAACAAATCCTGATCATCTAAAACAATATCAACATTCGTCATCCAGTACGAAACCTGCCAAAACTACGGAAGCTGAATCGACCAATAAAACTGCTAAAACTGCAGAAGC	

				AGAATCTACAACCAAACCGCCCTGCACATTTGG<br>TGCGAAATGTTATCGTACAAGTTCCATTCATTTA<br>GCTACTTATTCTCATCCATCTAAAAATGGAACAA<br>AGGAAGCGAGTGAAGAAGATATACTTGATACTC<br>GTGAATTAGTTGAAGCTGAAGAACCTGCTTCAC<br>CAACCACAACTGATAATTTATTAGTCAAAGATAA<br>AAATAAAGGCCTGACATTCGACGATGATGATGA<br>TGAAAGTAATGATCAAGAAATGGTTAGTCGTTC<br>TAAAAAAGATTGGACCAATCTTGAAAATAAATG<br>CAAAGAACAAAGCAAACGTTTAGCATATCTTGA<br>AGAAATGTTTAAAACGACAAAACGATCGAATTC<br>AACAACTGATAATAATGAAGAAAGCAACAAACG<br>TATGAAAACAGATTGAACGCGTGTCATGAGCT	
	DNA Ligase E_homoeo -log A	FUN_0191 85	Avaga_gblock_LIGEa	AGCTCATGACGCGATGAGTACAGCAGCAACACA<br>GGTTCTTCTTGCAGAAACATGGAGCGAAGATAT<br>CGATCCTACTGGTTGGTGGATGAGTGAAAAACT<br>TGATGGTGTTCGAGCGTATTGGAGTGGGAGTA<br>ACTTTTATTCTCGACAAGGAAATCTTTTTCACGT<br>GCCTGATTTTTTCAAGGCCGCACTTCCCAAAGTA<br>CCCTTGGATGGAGAAATATGGTGTGGTCGTGG<br>ACTCTTTCAAAAATGTATAAGTATTATAAAGAAA<br>CAGGGAAATAAAGTTGTTCCCGATGATTATAAG<br>CTCTTGACTTATTTGATTTTCGATGCACCAACTC<br>ATGGAGGAAAATATGAAGATCGTGTCAAATGG<br>TTACAGACGAATGTACCACAAGATGACGATAAA<br>TGTTATGCATCAGTTGTCGGTATAAAAAAATGTC<br>AAAATCTTGCAGATCTTAAACAATGTTTAGCTAC<br>TGTAAATGATGCCGGTGGAGAAGGAATTATGCT<br>CAGGAAACCTGGTAGTCTATATGAAAATAAGCG<br>TTCATCAACATTACTGAAAGTAAAAACGTTTTAT<br>GACGAAGAAGCTCTAGTTGTTGGTCATAAACCT<br>GGAAAAGGAAATTGTACAGGAGTTTTAGGTGCT<br>CTTCAATGTCAATTACCTAATGGAAAACGTTTCG<br>ATGTTGGAAGTGGCTTTGATATGTCTCAACGTC<br>GAAATCCACCTAAAAAAGGTTCAGTGATAACAT<br>TTAAATTTCAAGAATTATCTAATGCTGGTATTCC<br>ACGTTTTCCTGTATTTCTTCGCATTCGTAGTGATT<br>TAACTTGGAATGATGTTCTTGAAGCAGCTAAAA<br>CAAAAACACCAATAAGTACAACACAAAAAGTTG<br>TACCATCCGCAAAATTATCTAAGCAACATTCAAT<br>ATTATTCTCTATTATTCCATCACGTGATGTTAAA<br>ACTGGTAAGAAAGTTGTTGCATCCGATGAAGAA<br>GGTGAACAAATATCTTCGTCATCACCTTCGACAT<br>CAACTTCAAAAACGAAGACAGATAACCAAGAAG<br>TATGTCAATATGGAGCGAAATGTTATCGAACTA<br>ATGCCGATCACCTCAAACAATATCAACATCCAAC<br>ATCATCTAAATTAACGAAATCGAAATCGAAAAC<br>AAAATCATCTCCTACCTCTACATTAAAAATTACA<br>CGACAAAGTTCTAAGAAATCAGAATCTCAAGAT<br>GAACCAACTTCACCAACTATCACAAGTAATCTAC<br>TTGTCAAAGATAAAAATAAAGGTCGTCATTTCG	

				GTGACGATGATAAGGTAGATGATGATGATGAT GATGATGAACCAGCTGCAACACCTATAACAAGT AATAAACGAAAACGAAATGTTGTTGAAAAAAAA CCTGTTAGAAAACGTACAAAGCAATCTTAGACG CGTGTCATGAGCT	
	DNA Ligase 1_homoeo -log B	FUN_0117 91	Avaga_LIG1b_FW	TGACGATGAATCCGATCAAA	
			Avaga_LIG1b_RV	TCGAATGGTGAAAACAACGA	
	DNA Ligase 1_homoeo -log A	FUN_0117 93	Avaga_LIG1a_FW	CCATGTTAGCACATCCATCG	
			Avaga_LIG1a_RV	TGGTTGAAATGCATTCGGTA	
	DNA Ligase 3_homoeo -log A	FUN_0079 98	Avaga_LIG3a_FW	TTTCTTCATGCTCGTGCAAC	
			Avaga_LIG3a_RV	TCGCGGATTTAGTTGCTTTT	
	DNA Ligase 4_homoeo -log A	FUN_0237 39	Avaga_LIG4a_FW	CGTGCTGGTATGCTTTCTCA	
			Avaga_LIG4a_RV	TTCGACCATGGTTTCCATTT	
Adineta ricciae	*gapdh*	g37006	Aricciae_GAPDH_FW	CTGGTATGGCTTTCCGTGTT	2
			Aricciae_GAPDH_RV	GGCGTCGAAGATTGATGAGT	
	DNA Ligase E_homoe-log B	g12513	Aricciae_LIGEb_FW	ATGCTATGCGACAGTCGTTG	
			Aricciae_LIGEb_RV	TGACCAATGACGAGTGCTTC	
	DNA Ligase E_homoe-log A	g20670	Aricciae_LIGEa_FW	TGAAAGAGTCCTCGACCACA	
			Aricciae_LIGEa_RV	CTCGCAGAAACATGGAGTGA	
Mortie-rella verticilla-ta	*gapdh*	MVEG_052 52	Mverticillata_GAPDH _FW	TGAACACCCAGTGCATTCAT	3
			Mverticillata_GAPDH _RV	GGGCAAATATCGACAGGAAA	
	DNA Ligase E	MVEG_119 54	Mverticillata_LIGE_F W	AAGCCCTTCAACCTGTTCCT	
			Mverticillata_LIGE_R V	TGGGATGGCTAAATTGCTTC	
Homo sapiens	*gapdh*	*gapdh*	Hsapiens_GAPDH_F W	TGCACCACCAACTGCTTAG	4
			Hsapiens_GAPDH_F W	GTTCAGCTCAGGGATGACC	
n.a.	Oligos for *in vitro* BER assay (LIG03)	n.a.	n.a.	/5Phos/TCAACTCAGCAACTCCTT/3AmMO/	5
n.a.	Oligos for *in vitro* BER assay (Loop02)	n.a.	n.a.	/5FluorT/TTGGAGTTGCTGAGTTGATTCGTGAG CACCAACCGGTGCTCACGAA	

[1]Simion, P. *et al.* Chromosome-level genome assembly reveals homologous chromosomes and recombination in asexual rotifer Adineta vaga. *Sci Adv* 7, eabg4216 (2021).

[2]Nowell, R. W. *et al*. Comparative genomics of bdelloid rotifers: Insights from desiccating and nondesiccating species. PLoS Biology 16, e2004830 (2018).

[3]Vandepol, N. *et al*. Resolving the Mortierellaceae phylogeny through synthesis of multi-gene phylogenetics and phylogenomics. Fungal diversity 104, 267–289 (2020).

[4]Nicolas, E. *et al.* Involvement of human ribosomal proteins in nucleolar structure and p53-dependent nucleolar stress. Nature Communications 7, 11390 (2016).

[5]Healing, E. *et al*. A panel of colorimetric assays to measure enzymatic activity in the base excision DNA repair pathway. Nucleic Acids Research 47, e61–e61 (2019).

Source data files for supplementary information

Figure S1a

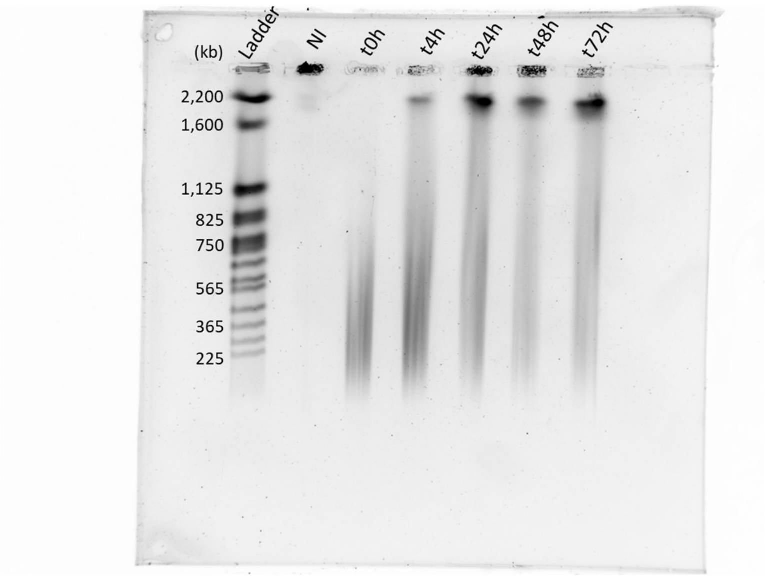

Figure S1b

The mass spectrometry proteomics data are available on the PRIDE partner repository (https://www.ebi.ac.uk/pride/).

Figure S1c

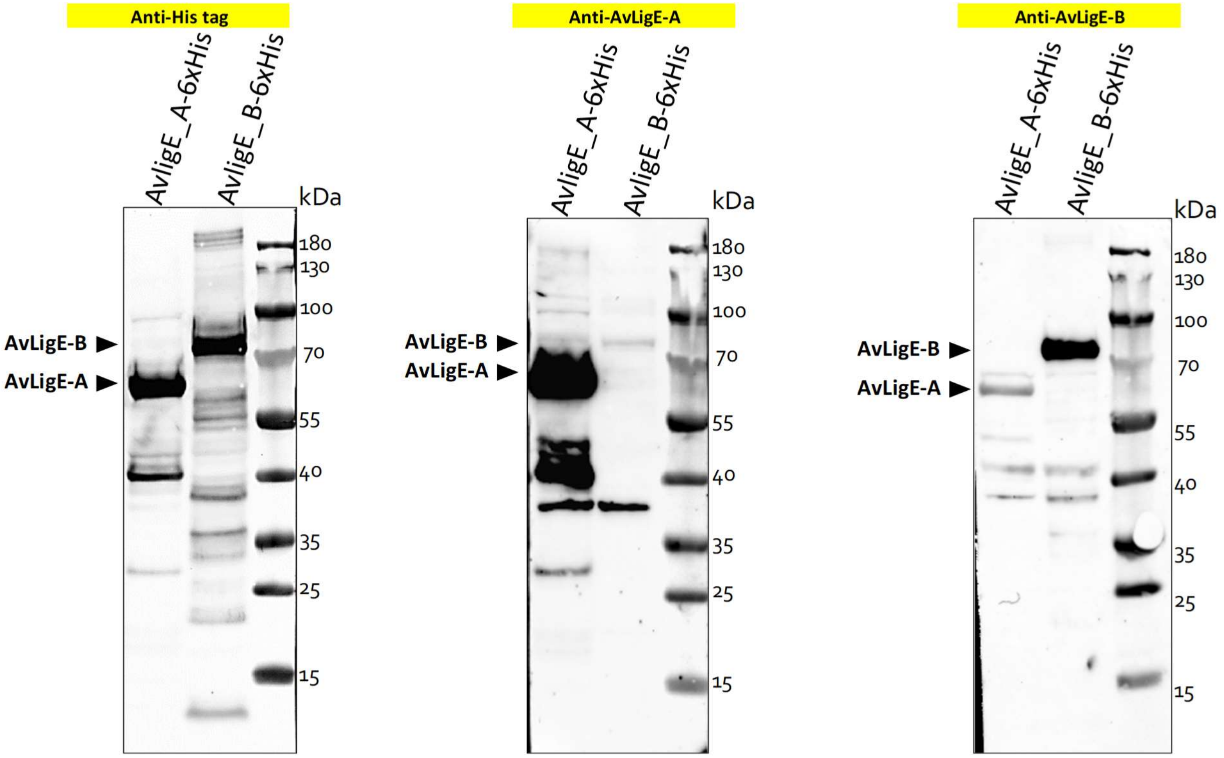

Figure S1d

Images presented on Figure S1d are the full images captured with Zeiss LSM 900 with Airyscan2 in Z-stacks. The insets are cropped regions from the full size images.

Figure S2

Complete sequence sets (i.e. raw, aligned and reduced) as well as the phylogenetic tree are available on Figshare: https://figshare.com/s/7cbed8c5d59abdd2f4ae.
