# Supplementary material for: Horizontal acquisition of a DNA ligase improves DNA damage tolerance in eukaryotes
Source: Nat Commun. 2023 Nov 22;14:7638. doi: 10.1038/s41467-023-43075-8 (PMC10665377; doi:10.1038/s41467-023-43075-8)
Supplement: Supplementary file 3 — Reporting Summary [file 41467_2023_43075_MOESM3_ESM.pdf]

Corresponding author(s): Nicolas Emilien, Hallet Bernard, Van Doninck Karine

Last updated by author(s): Oct 13, 2023

Reporting Summary

Nature Portfolio wishes to improve the reproducibility of the work that we publish. This form provides structure for consistency and transparency in reporting. For further information on Nature Portfolio policies, see our Editorial Policies and the Editorial Policy Checklist.

Statistics

For all statistical analyses, confirm that the following items are present in the figure legend, table legend, main text, or Methods section.

n/a	Confirmed	
☐	☒	The exact sample size (*n*) for each experimental group/condition, given as a discrete number and unit of measurement
☐	☒	A statement on whether measurements were taken from distinct samples or whether the same sample was measured repeatedly
☐	☒	The statistical test(s) used AND whether they are one- or two-sided<br>*Only common tests should be described solely by name; describe more complex techniques in the Methods section.*
☒	☐	A description of all covariates tested
☐	☒	A description of any assumptions or corrections, such as tests of normality and adjustment for multiple comparisons
☐	☒	A full description of the statistical parameters including central tendency (e.g. means) or other basic estimates (e.g. regression coefficient) AND variation (e.g. standard deviation) or associated estimates of uncertainty (e.g. confidence intervals)
☐	☒	For null hypothesis testing, the test statistic (e.g. *F*, *t*, *r*) with confidence intervals, effect sizes, degrees of freedom and *P* value noted<br>*Give P values as exact values whenever suitable.*
☒	☐	For Bayesian analysis, information on the choice of priors and Markov chain Monte Carlo settings
☒	☐	For hierarchical and complex designs, identification of the appropriate level for tests and full reporting of outcomes
☒	☐	Estimates of effect sizes (e.g. Cohen's *d*, Pearson's *r*), indicating how they were calculated

Our web collection on statistics for biologists contains articles on many of the points above.

Software and code

Policy information about availability of computer code

Data collection	- Mass spectrometry raw and processed data have been submitted to the PRIDE (Proteomics Identifications Database) repository<br>- DNA LigE subcellular localization images were captured using Zeiss LSM 900 using AiryScan2 with constant parameters<br>- Horizontal gene transfer level was determined using Alienomics, a custom computer pipeline described in Simion et al., 2021, doi. 10.1126/sciadv.abg4216<br>- For the phylogenetic analysis, we used diamond BLASTp (Buchfink et al., 2021, doi. 10.1038/s41592-021-01101-x), Bedtools (Quinlan & Hall, 2010, doi. 10.1093/bioinformatics/btq033), mafft (Katoh et al., 2002, doi 10.1093/nar/gkf436), and iq-tree 1.6.10 (Nguyen et al., 2015, doi. 10.1093/molbev/msu300)<br>- Conserved protein domains were predicted using InterPro (Blum et al., 2020, doi. 10.1093/nar/gkaa977)<br>- qPCR data were collected using the software from Applied Biosystems 7500 instrument
Data analysis	- Microscope images were analyzed using zen 3.4 (blue edition) or Fiji software (v2.1.0/1.53c)<br>- Proteomic data analysis was performed using PEAKS Studio X+<br>- Mounting and analysis of the images were done with the Fiji software (v2.1.0/1.53c)<br>- Visual inspections of phylogenetic trees were performed with Archaeopterix (Han & Zmasek, 2009, doi. 101186/1471-2105-10-356) and iTol (Letunic & Bork, 2021, doi. 10.1093/nar/gkab301)<br>- qPCR and in vitro DNA ligation data were analyzed with GraphPad Prism 7 softwareand related statistical analysis were also performed using GraphPad Prism 7 software

For manuscripts utilizing custom algorithms or software that are central to the research but not yet described in published literature, software must be made available to editors and reviewers. We strongly encourage code deposition in a community repository (e.g. GitHub). See the Nature Portfolio guidelines for submitting code & software for further information.

Data

Policy information about availability of data

All manuscripts must include a data availability statement. This statement should provide the following information, where applicable:

- Accession codes, unique identifiers, or web links for publicly available datasets
- A description of any restrictions on data availability
- For clinical datasets or third party data, please ensure that the statement adheres to our policy

Raw data are available in the Source Data file related to this manuscript. Protein sequences used in this study are available in the public repository NCBI NCBI, assembly ASM2161353v1. Mass spectrometry raw and processed data have been submitted to the PRIDE (Proteomics Identifications Database) repository with the dataset identifier PXD043051. Datasets used for phylogenetic analyses are available on Figshare: https://figshare.com/s/7cbed8c5d59abdd2f4ae. Data presented in this manuscript are protected under the patent WO 2023/161266 Methods and Compositions for increasing stress tolerance of cells and organisms (wipo.int).

Research involving human participants, their data, or biological material

Policy information about studies with human participants or human data. See also policy information about sex, gender (identity/presentation), and sexual orientation and race, ethnicity and racism.

Reporting on sex and gender	n.a.
Reporting on race, ethnicity, or other socially relevant groupings	n.a.
Population characteristics	n.a.
Recruitment	n.a.
Ethics oversight	n.a.

Note that full information on the approval of the study protocol must also be provided in the manuscript.

Field-specific reporting

Please select the one below that is the best fit for your research. If you are not sure, read the appropriate sections before making your selection.

☒ Life sciences ☐ Behavioural & social sciences ☐ Ecological, evolutionary & environmental sciences

For a reference copy of the document with all sections, see nature.com/documents/nr-reporting-summary-flat.pdf

Life sciences study design

All studies must disclose on these points even when the disclosure is negative.

Sample size	The number of animals (bdelloid rotifer Adineta vaga) processed from each experiment was determined according to the follow-up experiment. Particularly, for mass spectrometry analysis, we processed a large quantity of animals to be able to process at least 50 micrograms of each protein extract. Similarly, the number of animal processed during the immuno-localization experiments was optimal to be able to retrieve enough rotifers on the slide for the final microscopy analysis. The quantity of human cells processed for the colony formation assay is standard (e.g. described in Franken et al., 2006, doi. 10.1038/nprot.2006.339).
Data exclusions	No data were excluded from the analysis
Replication	- Each comparative proteomic analysis (at 0.8 kGy and 1.0 kGy) were performed from two independent biological replicates and from two MS injections per sample. Knowing that the bdelloid rotifer populations are clonal and grown under constant culture conditions and treated with a carefully calibrated irradiation facility, we consider that the number and diversity of processed samples is high enough for the purpose of this study. Results using individual samples were comparable to the results obtained with the pooled replicates indicating that the replication was successful.<br>- The number of samples and replicates considered for the other experiments, such as the transcription level assessment by qPCR, the in vitro DNA ligation assay or the colony formation assay (from 3 to 6) are standard for this kind of assay. The narrow distribution of individual data shows that the replication was successful.
Randomization	This study did not involve any randomization procedure as it used clonal populations of bdelloid rotifers are human cells.
Blinding	This study did not involve any blinding procedure as it used clonal populations of bdelloid rotifers are human cells.

Reporting for specific materials, systems and methods

We require information from authors about some types of materials, experimental systems and methods used in many studies. Here, indicate whether each material, system or method listed is relevant to your study. If you are not sure if a list item applies to your research, read the appropriate section before selecting a response.

Materials & experimental systems

n/a	Involved in the study
☐	☒ Antibodies
☐	☒ Eukaryotic cell lines
☒	☐ Palaeontology and archaeology
☐	☒ Animals and other organisms
☒	☐ Clinical data
☒	☐ Dual use research of concern
☒	☐ Plants

Methods

n/a	Involved in the study
☒	☐ ChIP-seq
☒	☐ Flow cytometry
☒	☐ MRI-based neuroimaging

Antibodies

Antibodies used	- Custom polyclonal antibodies targeting AvLigE-A/B copies were synthesized against C-terminal peptides of the proteins by Eurogentec<br>- Anti-tubulin primary antibody (Sigma Aldrich, ref. T6199)<br>- Anti-histone H3 antibody (Bethyl laboratories, ref. 50-155-8538)<br>- Anti-His tag antibody (ThermoFisher Scientific, ref. MA1-21315)<br>- Anti-rabbit coupled to HRP antibody (ThermoFisher Scientific, ref. A16116)<br>- Anti-mouse coupled to HRP antibody (ThermoFisher Scientific, ref. 31431)<br>- Alexa 568 Goat anti-rabbit antibody (ThermoFisher Scientific, ref. A11011)
Validation	- The custom polyclonal antibody targeting AvLigE-A/B specificity and efficiency was assessed by comparison of the signal and expected size of the protein obtained in a Western-blot using various dilutions of the antibody or of the pre-immune sera. The specificity of the antibody was also assessed by showing that the immuno-depletion of AvLigE-B by the custom antibody was specifically removing AvLigE-B protein. Also, when AvLigE-A/B proteins were fused with a C-terminal His-tag, the Western-blots show a similar profile by using anti-AvLigE-A/B antibodies than by using anti-His antibody, validating the results of the blots (Figure S1c).<br>- The commercial antibodies (primary and secondary) were used following the manufacturer's instructions and recommended dilutions. All commercial antibodies used in this study followed an enhanced-validation step to ensure antibody reproducibility, specificity and performance.

Eukaryotic cell lines

Policy information about cell lines and Sex and Gender in Research

Cell line source(s)	HEK 293 T cell line (Homo sapiens, epithelial, kidney) was purschased from ATCC (ref. CRL-3216)
Authentication	The original HEK 293 T cell line was not authenticated. However, the parental cell line was modified by CRISPR technology using Stable cell line AVVS1 kit (Origene) to stably insert and express the AvLigE-A/B copies. The insertion site and the identity of the sequences were verified by sequencing of the genomic region surrounding the insertion site.
Mycoplasma contamination	The cell lines were not tested for mycoplasma contamination
Commonly misidentified lines<br>(See ICLAC register)	HEK 293 T is not a commonly misidentified cell line

Animals and other research organisms

Policy information about studies involving animals; ARRIVE guidelines recommended for reporting animal research, and Sex and Gender in Research

Laboratory animals	All experiments were performed using isogenic Adineta vaga clones derived from a single individual from Meselson Laboratory. Heterogenous populations of bdelloid rotifers of different ages were used. The mean life span of bdelloid rotifer under this constant condition is between 30-40 days.
Wild animals	No wild animals were used in this study
Reporting on sex	n.a.
Field-collected samples	No filed-collected samples were used in this study

Ethics oversight	This study did not require an ethical approval

Note that full information on the approval of the study protocol must also be provided in the manuscript.

Plants

Seed stocks	n.a.
Novel plant genotypes	n.a.
Authentication	n.a.
